# Potent anticancer activity of cystine-based dipeptides and their interaction with serum albumins

**DOI:** 10.1186/1752-153X-7-91

**Published:** 2013-05-24

**Authors:** Biswadip Banerji, Sumit Kumar Pramanik, Uttam Pal, Nakul Chandra Maiti

**Affiliations:** 1 Department of Chemistry, CSIR-Indian Institute of Chemical Biology, 4, Raja S.C. Mullick Road, Kolkata 700032, India; 2 Department of Structural Biology and Bioinformatics, CSIR-Indian Institute of Chemical Biology, 4, Raja S.C. Mullick Road, Kolkata 700032, India

**Keywords:** Peptide, Anticancer, Serum albumin, Spectroscopy, Docking

## Abstract

**Background:**

Cancer is a severe threat to the human society. In the scientific community worldwide cancer remains a big challenge as there are no remedies as of now. Cancer is quite complicated as it involves multiple signalling pathways and it may be caused by genetic disorders. Various natural products and synthetic molecules have been designed to prevent cell proliferation. Peptide-based anticancer drugs, however, are not explored properly. Though peptides have their inherent proteolytic instability, they could act as anticancer agents.

**Results:**

In this present communication a suitably protected cystine based dipeptide and its deprotected form have been synthesized. Potent anticancer activities were confirmed by MTT assay (a laboratory test and a standard colorimetric assay, which measures changes in colour, for measuring cellular proliferation and phase contrast images. The IC_50_ value, a measure of the effectiveness of a compound in inhibiting biological or biochemical function, of these compounds ranges in the sub-micromolar level. The binding interactions with serum albumins (HSA and BSA) were performed with all these molecules and all of them show very strong binding at sub-micromolar concentration.

**Conclusions:**

This study suggested that the cystine-based dipeptides were potential anticancer agents. These peptides also showed very good binding with major carrier proteins of blood, the serum albumins. We are currently working on determining the detailed mechanism of anticancer activity of these molecules.

## Introduction

**Figure 1:**
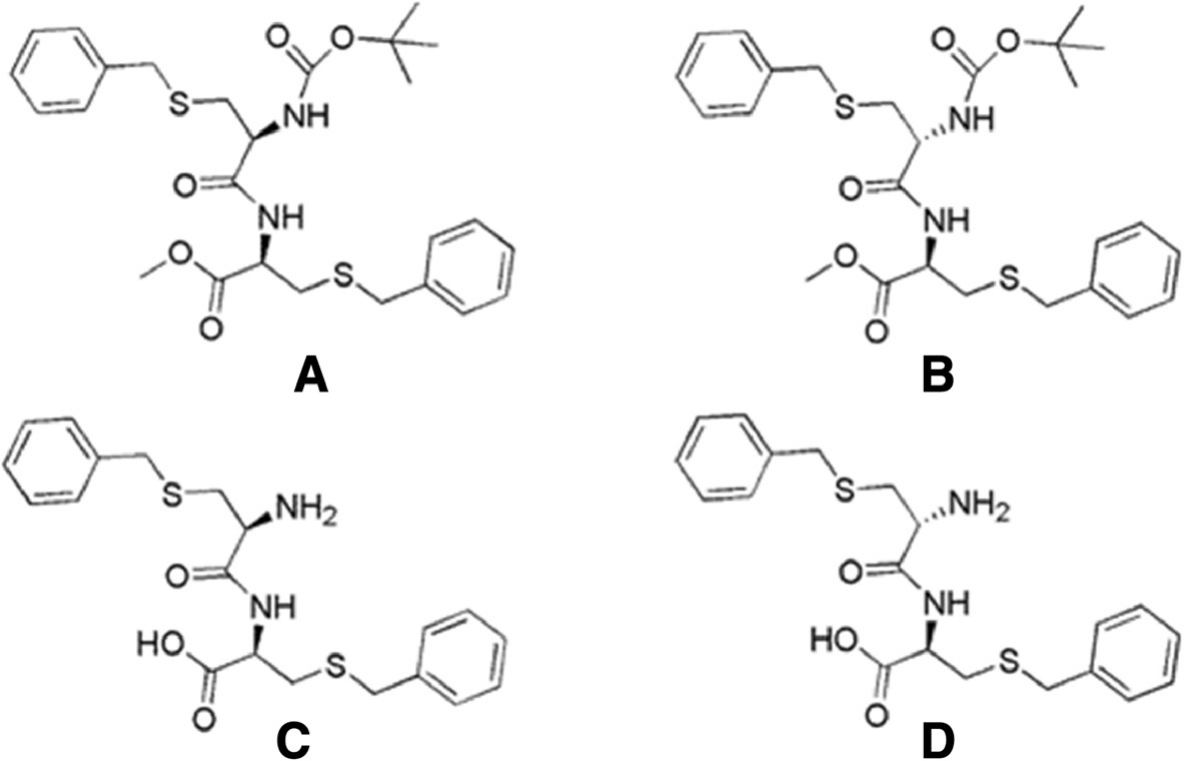
**The structures of cysteine derived dipeptide compounds.** Where **A** corresponds to 1**A**, **B** corresponds to 1**B**, **C** corresponds to 1**C** and **D** corresponds to 1**D** respectively.

Cancer has been an ever-growing public problem since its appearance and the estimated worldwide new incidence of it is about 6 million cases per year [1-4]. It is the second major cause of death after cardiovascular disease [5]. This disease is now well characterized by unregulated proliferation of cells [6,7]. There has long been a search for a therapeutic agent to inhibit or control cell proliferation. Various natural products along with synthetic molecules are continuously explored to achieve development of a viable anticancer molecule [8-10]. Peptides are very versatile biological molecules. Except for a few inherent problems, peptides or peptide-based molecules are most bio-compatible [11,12]. Compared with traditional treatments such as chemotherapy, peptides with high specificity against cancer cells may present an alternate way of killing cancer cells while protecting normal cells [13]. Many natural or synthetic peptides have been reported to show anticancer activity [14]. Peptide-based (or peptide-derived) anticancer drugs have the potential to selectively target and disrupt the signalling pathways in the course of carcinogenesis [15]. In the present study, we have synthesized a few cystine based dipeptide compounds (protected and deprotected L-Cys-L-Cys, L-Cys-D-Cys, 1A**-**1D) (Figure 1). These compounds show anticancer activity against different cancer cell lines. In addition, we performed an interaction study of them with serum albumins. In blood all the drugs must bind with the serum albumins in order to reach the target site [16-18]. Therefore, the study of serum protein binding with a newly synthesized drug molecule is very important [19-21]. In this paper we wish to disclose the sub-micromolar anticancer activity of these peptides and their binding interactions with serum albumins (BSA and HSA). Cell viability assay was done by MTT-assay while the binding studies were carried out using fluorescence spectroscopy, circular diachroism, molecular modelling and computational analysis. The low-micromolar anticancer activity may be further improved by changing different protection groups.

## Experimental methods

### Cell culture

Neura 2a (neuroblastoma cell line), Hek 293 (kidney cancer cell line) and Hep G2 (liver cancer cell line) were procured from the National Centre for Cell Sciences (NCCS, Pune, India) and were grown in Dulbecco’s modified Eagle medium antibiotics (penicillin/streptomycin and gentamicin). Cells were cultured at 37**°**C in 95% air and 5% CO_2_ humidified incubators. Hep G2 cells were seeded at a density of 10^5^ well plated in 96 well plates. Cells were typically grown to 60–70% confluence, rinsed in phosphate-buffered saline (PBS) and placed into serum-free medium overnight prior to treatments. After overnight incubation, the Hep G2, HEK 293, and Neura 2a cells were treated with these compounds separately at the concentration of 1 μM, 10 μM and 20 μM, respectively. After 48 hours the medium was removed and a 50 μl of fresh medium was added along with 10 μl of MTT (3-(4,5-Dimethylthiazol-2-yl)-2,5-diphenyltetrazolium bromide). MTT solution (5mg/ml) was slowly removed after 4 hours and the purple crystals with solubilization in 1.4 ml of DMSO. The absorbance was measured at test wavelength of 550 nm in Elisa Plate Reader [22,23].

### Fluorescence

The steady-state fluorescence spectra were recorded with a Perkin Elmer LS-45 spectrofluorophotometer. Emission spectra were recorded with an excitation wavelength of 280 nm and emission range of 290–450 nm. Both the excitation and emission slit widths kept at 5 nm each. The intrinsic fluorescence of tryptophan residue(s) in the protein was measured in the presence and in the absence of the dipeptides. Most of the experiment was carried out at room temperature (25°C), Some temperature dependent studies were carried out using water bath.

The fluorescence of the protein was found to quench in the presence of the peptides. The quenching experiment was carried out simply by adding small aliquote (1–10 μL from 100 μM stock solution) of concentrated peptide solution to 1 mL solution containing an appropriate concentration of HSA/BSA (0.5 μM in 20 mM Tris–HCl buffer, pH 7.5) taken in 1 cm path length quartz cuvette. The optical density of the solution at the excitation wavelength was kept less than 0.05. Small error due to dilution upon addition of the peptide was neglected. The peptides showed negligible absorbance at the excitation wavelength (280 nm). Fluorescence intensities at 340 nm were recorded as a function of ligand concentration. To derive the binding parameters, obtained data were analyzed using modified Stern–Volmer equation [24-26].

### Measurements of circular dichroism (CD)

The Far-UV CD spectra have been measured on a Jasco J-810 spectrometer using a 1.0 mm quartz cell under constant nitrogen flow condition and at room temperature. The CD spectra of HSA and BSA have been recorded in the absence and presence of these compounds within the wavelength range of 200–250 nm. The CD results have been represented in terms of ellipticity (θ).

### Docking

The crystal structure of HSA and BSA were obtained from Protein Data Bank (PDB ID: 1E78 and 3V03 respectively). Structures of the synthesized compounds were drawn in Gauss View followed by geometry optimization in Gaussian 09 with DFT level of theory using B3LYP/6-31 + G(d,p) basis set. AutoDock 4 and MGLTools of The Scripps Research Institute were used to perform the docking calculations [27,28]. Docking was performed following the previously published protocol [29-33]. The PyMOL molecular (http://pymol.org/) viewer and the MGLTools were used to render the output.

## Results and discussion

### Cell viability

In order to determine the biological efficacy of these newly synthesized compounds in vitro cell culture system has been used.

Cell viability was quantified by MTT, a yellow tetrazole assay, where the viable cells were determined by the reduction of the yellow MTT into purple formazan product. For this assay, the cells were plated in 96 well plates and grown in monolayer and then treated with these compounds of interest. The viability of cells by MTT assay was performed 48 hours post treatment as described before. Finally, the medium was removed and replenished with 80 μl of fresh medium along with 20 μl of MTT (5 mg/ml). After 4 hours, MTT solution was slowly removed and the purple crystals were solubilised in 100 μl of DMSO. The absorbance was measured by a plate reader at a wavelength of 550 nm. The absorbance obtained from treated cells were expressed as percentages of absorbance obtained from untreated cells and are reported as mean ± SEM (*n* =3).

**Figure 2:**
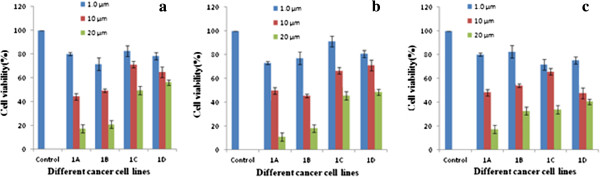
Cytotoxicity studies against Neura 2a (2a), Hep G2 (2b), Hek 293 (2c), cell lines presented respectively.

For screening the activity, the cultured cells were exposed to these compounds at three different concentrations (1.0 μM, 10 μM and 20 μM) and incubated for 48 hours. Viability was assessed by MTT assay as described. All the four compounds showed significant reduction in the amount of viable cells in all the three cell lines screened. The results are shown graphically below, Figure 2a**-**c, respectively. From the bargraph it is observed that these peptides cause significant reduction of viable cells in this screening assay.

**Figure 3:**
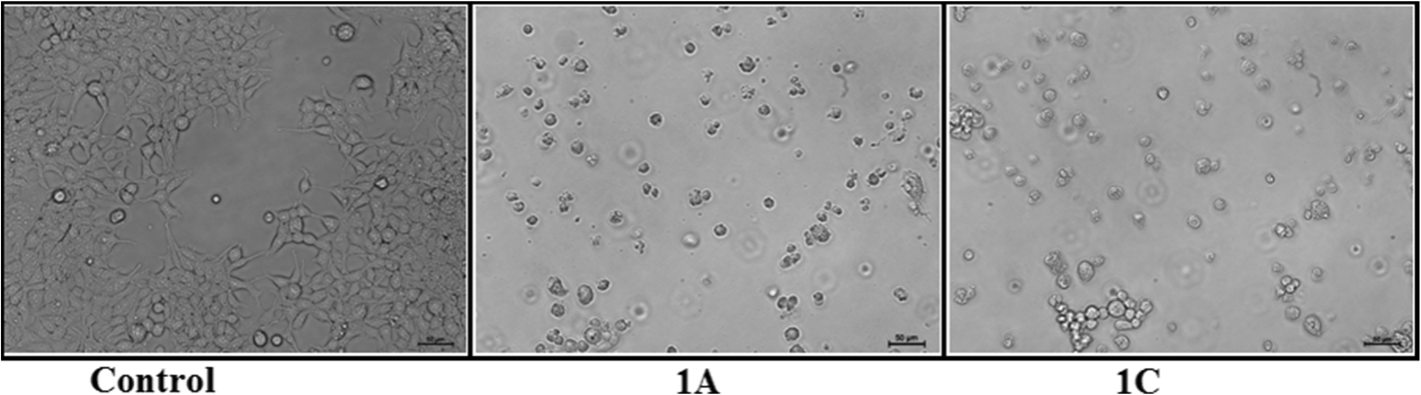
Phase contrast images showing cell death with compounds 1A and 1C at 20.0 μM concentration.

The compounds 1A and 1B show more cytotoxicity than compounds 1C and 1D at a particular concentration. Cytotoxiciy of 1A is comparable to 1B and the cytotoxicity of 1C is comparable to 1D. Furthermore, cells were also examined under an inverted phase contrast microscope. For example, Hek 293 cells were treated with these compounds (at 20.0 μM concentration) for 24 hours and phase contrast micrographs were taken. As shown in Figure 3, there was massive cell death in response to these two compounds (1A and 1C) as compared to control.

Action of a drug molecule to a cell is initiated by drug receptor and many of the receptors have high specificity for a drug molecule and the chemical structure of a drug may significantly alter the cell's response to the drug molecule. Also the concentration of drug molecule to the receptor site directly affects the drug response. For example, amphetamine and methamphetamine act as powerful stimulus for nervous system and act via the same receptor. These two compounds differed slightly in their chemical structure; however, methamphetamine exerts more powerful action. There are small structural changes present in our synthesized dipeptides. NH_2_ groups in 1A and 1B are protected with carbamates, also the carboxylic acid moiety is as a methyl ester. The receptor that initiates the drug action of the dipeptides may show difference in action due to these structural changes. However, similar to many chemical reactions, drug action of the receptor also depends on the effective concentration of the drug molecule at the receptor site. Amount of drug that penetrates to the cell/receptor site again depends on structure of the drug molecule and their physical parameter such as hydrophobicity. One possible explanation is that 1A and 1B (cLogP: 4.01, see Additional file 1: Computation of partition coefficient (cLogP)) are more hydrophobic than 1C and 1D (cLogP: 1.75). So, the membrane permeability of these two are more than the other two. So, 1A and 1B can penetrate the cells better than that of 1C and 1D and could be sensed by the receptor more strongly apart from the structural specificity.

Cell viability tests were performed using cultured cells. However, in real systems, like cells in human body/other animals drugs need to be reached to the body/effected cells by blood. All the drug molecules that enter into the body via systemic circulation get exposed to the blood milieu. In blood, serum protein albumins (HSA, BSA) are the major carrier proteins. They bind to a wide variety of small molecules and fatty acids and carry of them to different parts of the body. Very good binding to these proteins means very good distribution of the drug all over the body i.e., increased bioavailability. Therefore, the binding behaviour of the synthesized peptides to HSA and BSA was carried out using the unique and intrinsic fluorescence from the tryptophan residues. The dipeptides showed very good binding with plasma carrier proteins of both bovine and human. Interaction site of the peptides to the protein was established via molecular docking analysis as discussed later.

### Binding constant from fluorescence study

**Figure 4:**
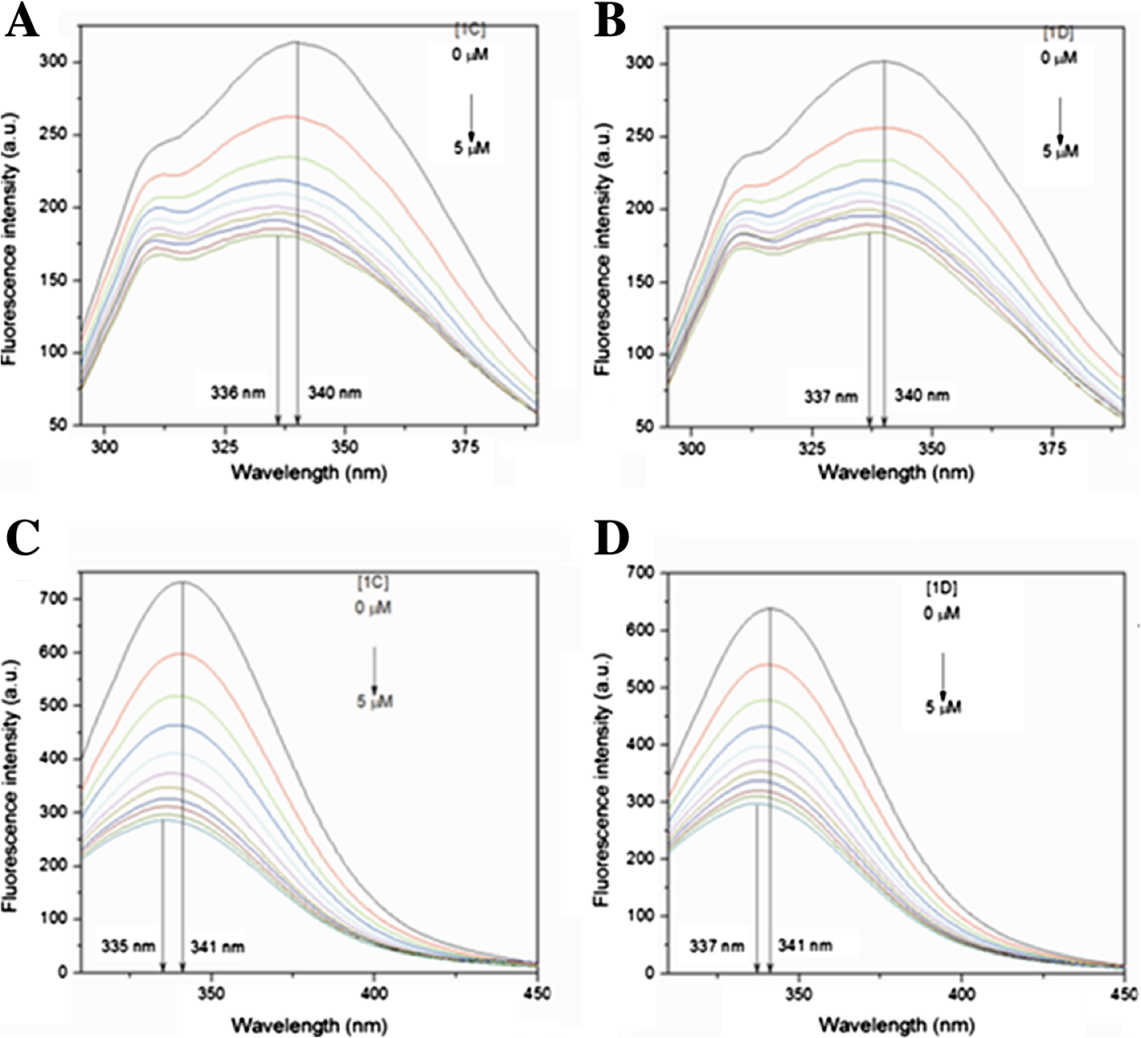
**Effect of the compounds on the intrinsic fluorescence of serum albumins.** Here **A** and **B** are Emission spectra of HSA as a function of compound 1**C** and 1**D** respectively with concentration varying from 0 μM to 5 μM (10 steps). Figure **C** and **D** are Emission spectra of BSA as a function of compound 1**C** and 1**D** respectively with concentration varying from 0 μM to 5 μM (10 steps). About 4 to 6 nm blue shift in emission maximum was observed. Excitation maximum, 280 nm; excitation and emission slit 5 nm each.

The fluorescence spectra of HSA / BSA were measured in the presence and absence of cystine based dipeptide compounds. HSA shows a strong fluorescence with a emission peak at ~340 nm due to its single tryptophan residue (Figure 4). BSA with two tryptophan residues showed similar fluorescence behavior, however, with higher intensity due to the presence of two tryptophan residues in BSA. Cystine based dipeptides (1A, 1B, 1C and 1D**)** showed no intrinsic fluorescence in solution. However, their (compounds 1A, 1B, 1C and 1D**)** individual presence in the solution effectively reduced fluorescence yield of HSA / BSA (slight blue shift, ~ 4 nm of the fluorescence emission, was within the band width of the measurement). The fluorescence intensity at 340 nm decreased gradually with increasing peptide concentration, indicating effective fluorescence quenching of the protein fluorescence. Figure 4 shows the spectra in the presence of different concentrations of these dipeptide compounds 1C and 1D with HSA and BSA respectively. The quenching spectra for 1A and 1B with HSA and BSA are shown in Additional file 1: Figure S1.

Fluorescence data in the above experiments can be analyzed using a modified Stern-Volmer (S-V) equation [24] (equation 1). Fluorescence peak intensity values of the protein at different concentration of the compounds were used to fit a modified S-V equation as given below:

(1) $$\frac{F_{0}}{\mathrm{\Delta F}}=\frac{1}{\mathrm{fK}\left\lfloor Q\right\rfloor }+\frac{1}{f}$$

**Figure 5:**
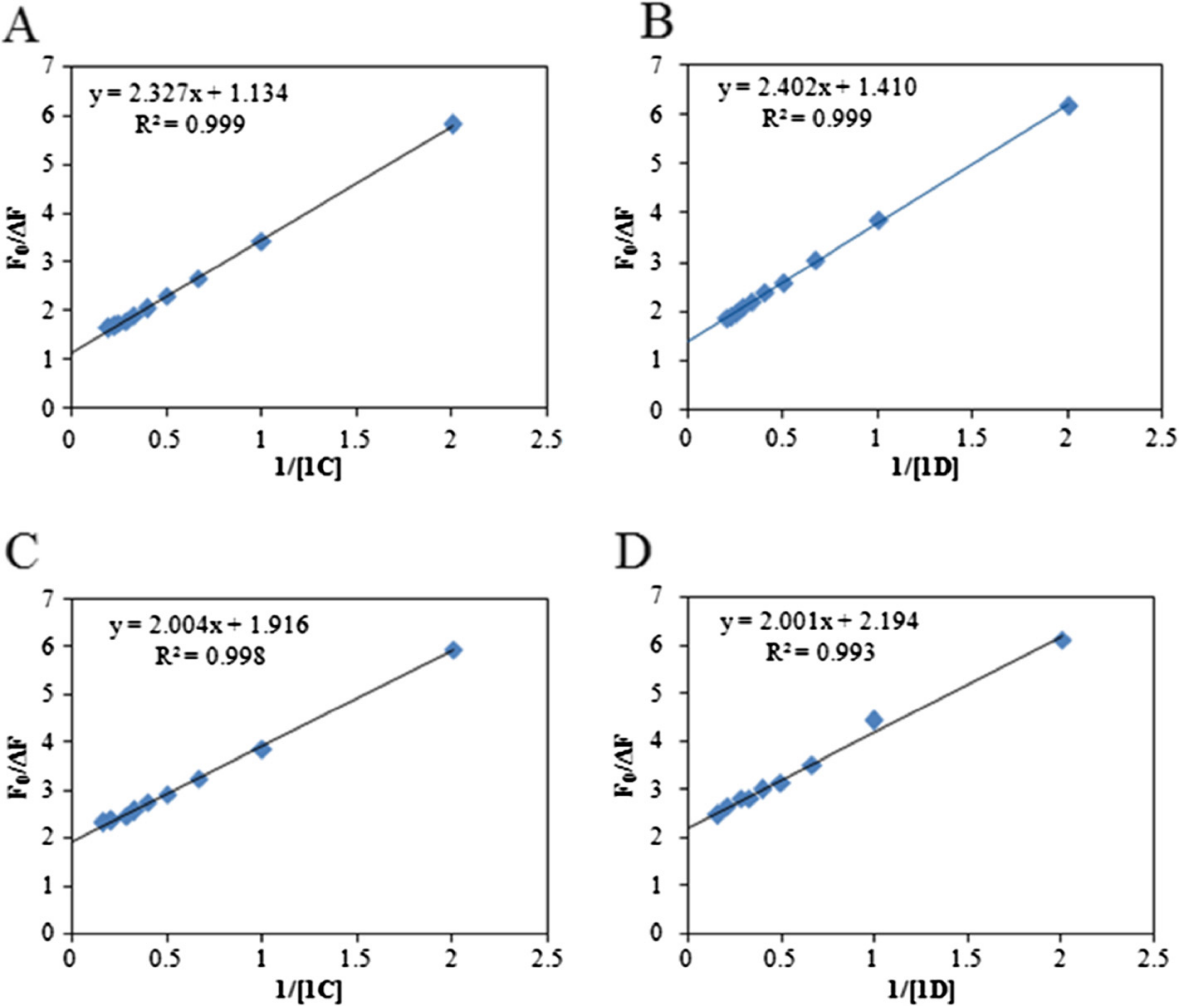
**Modified Stern-Volmer plot.** (**A**) and (**B**) for BSA with compounds 1**C** and 1**D**, respectively. (**C**) and (**D**) for HSA with compound 1**C** and 1**D**, respectively.

Where *F*_0_ is the fluorescence intensity in the absence of an external quencher, Δ*F* is the difference in fluorescence in the absence and presence of the quencher at concentration [*Q*], *K* is the Stern–Volmer quenching constant, and *f is* the fraction of the initial fluorescence which is accessible to the quencher. The plots of *F*_0_/Δ*F* versus 1 / [*Q*] (Figure 5) yields f ^−1^ as the intercept, and (f K)^−1^ as the slope. Table 1 shows the result. The intercept on y axis (f^-1^) indicated that ~70-90% of the total HSA fluorescence and ~50% of BSA fluorescence was accessible for the quenchers (dipeptides). It also suggests that only one tryptophan of BSA was accessible to the quencher. Further temperature dependent experiment showed that quenching constant for all the dipeptides decreased with increasing of temperature (Additional file 1: Table S1). This fact implied that the fluorescence quenching of the protein solution by the peptides was dominated by static quenching mechanism [24-26]. High accessibility of the quencher and the decrease of quenching constant indicated static fluorescence quenching and this static quenching arose from the formation of a dark complex between protein and dipetides [24,25]. The S-V quenching constant as obtained from the modified S-V equation can be shown to be the binding affinity constant, K_a_ (Additional file 1: Fluorescence Study) [24]. Reciprocal of this K_a_ gives the dissociation constant, K_d_ (Table 2). Contribution of dynamic quenching due to diffusion and collision of the peptides might be negligible as we observed that accesiblity of the peptides to the fluorphore (tryptophan residue in the protein) was high (70-90% for HSA and ~ 50% as explained earlier). We observed negligible amount of fluorescence quenching of free tyrptophan in the presence of the dipeptide solution (Additional file 1: Figure S3). This observation added additional support that the quenching of the protein fluorescence by dipeptides was largely due to association of the dipeptides close to the tryptophan residue in the protein. It supported the view that the peptides may be incorporated close to the tryptophan residue in the proteins and formed a close association (dark complex) and quenched fluorescence [26].

**Table 1:** Stern-Volmer quenching constant (K) with HSA and BSA at temperature 298 K as obtained from equation 1

**Compounds**	**Stern-Volmer quenching constant (M**^**-1**^**)**	
	**HSA**	**BSA**
1A	18.37 × 10^5^	7.95 × 10^5^
1B	4.32 × 10^5^	3.52 × 10^5^
1C	9.56 × 10^5^	4.88 × 10^5^
1D	10.97 × 10^5^	5.90 × 10^5^

**Table 2:** **Binding dissociation constants (K**_**d**_**) with HSA and BSA at temperature 298 K**

**(SEM = standard error of mean; NA = not available) Compounds**	**Binding constant (K**_**d**_**) ± SEM in μM**	
	**HSA**	**BSA**
**1A**	0.546 ± 0.05	1.257 ± NA
**1B**	2.312 ± NA	2.840 ± NA
**1C**	1.044 ± 0.08	2.051 ± NA
**1D**	0.912 ± NA	1.703 ± 0.07

There is the same relationship between 1A and 1B that between 1C and 1D: diastereoisomers. However, all the four compounds showed similar binding efficiency (Tables 1 and 2). It indicated that both the conformations are equally significant in the attenuation of HSA/BSA fluorecence. Eftink *et. al.* and others clearly indicated how the quenching volume and the entry of the quencher to the hydrophobic protein pocket influence both the static and dynamic quenching [24-26]. As in this investigation no significant difference occurred in quenching efficiency (quenching / binding constant), the dipeptides had similar accessibility of the tryptophan residues in the proteins.

### Circular dichroism (CD)

**Figure 6:**
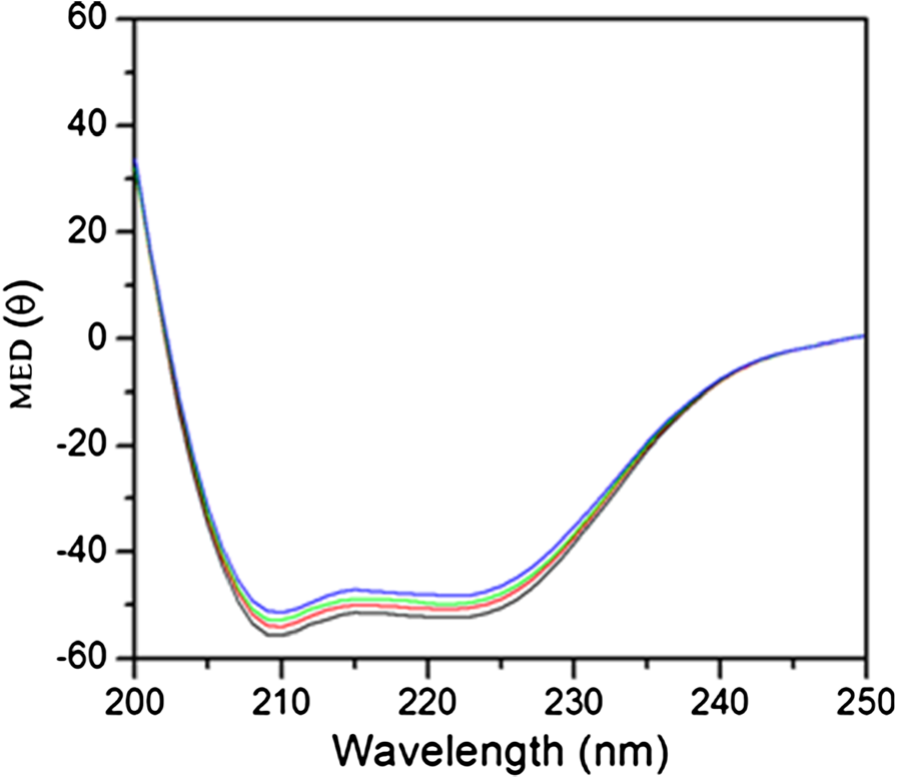
**Representative circular dichroism spectra of HSA in presence of different concentrations of compound 1A.** The black color line indicates the spectrum of HSA (4 μM), the red, green and blue color line indicate the spectrum of HSA after addition of 4 μM, 8 μM and 16 μM of compound 1A respectively.

The effect of binding of these compounds on the secondary structure of the protein has been determined through far-UV circular dichroism (CD). The CD spectra for HSA (Figure 6) observed in the range 200–250 nm reveal the presence of two bands at ~209 nm and ~222 nm, typically characteristic of α-helicity as consistent with the literature. Binding of the compounds (1A, 1B, 1C and 1D) to the proteins resulted in slight change in the secondary structure of the protein as evident from the change in the CD spectra (Figure 6).

**Figure 7:**
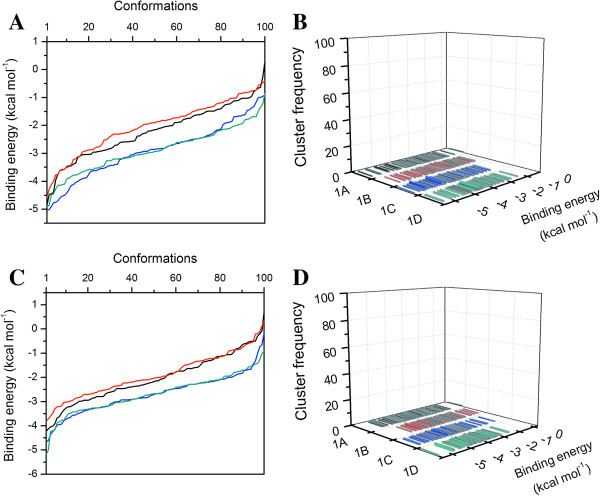
**Energy spectrum distribution of the bound conformations for HSA (A) and BSA (C).** All the best output from 100 independent docking simulations were arranged according to their binding energy. Very low energy conformations indicate thermodynamically favorable interaction. Binding of 1**C** and 1**D** with both the HSA and BSA were found to be better than that of 1**A** and 1**B**. But the inconsistency in the low energy binding modes suggests nonspecificity. Clustering of the bound conformations for HSA (**B**) and BSA (**D**). Binding modes within 2 Å standard deviation and 0.5 kcal mol^-1^ energy tolerance levels were clustered together. Although the low energy binding modes are prevalent no significant clustering was observed again suggesting no specificity in binding.

**Figure 8:**
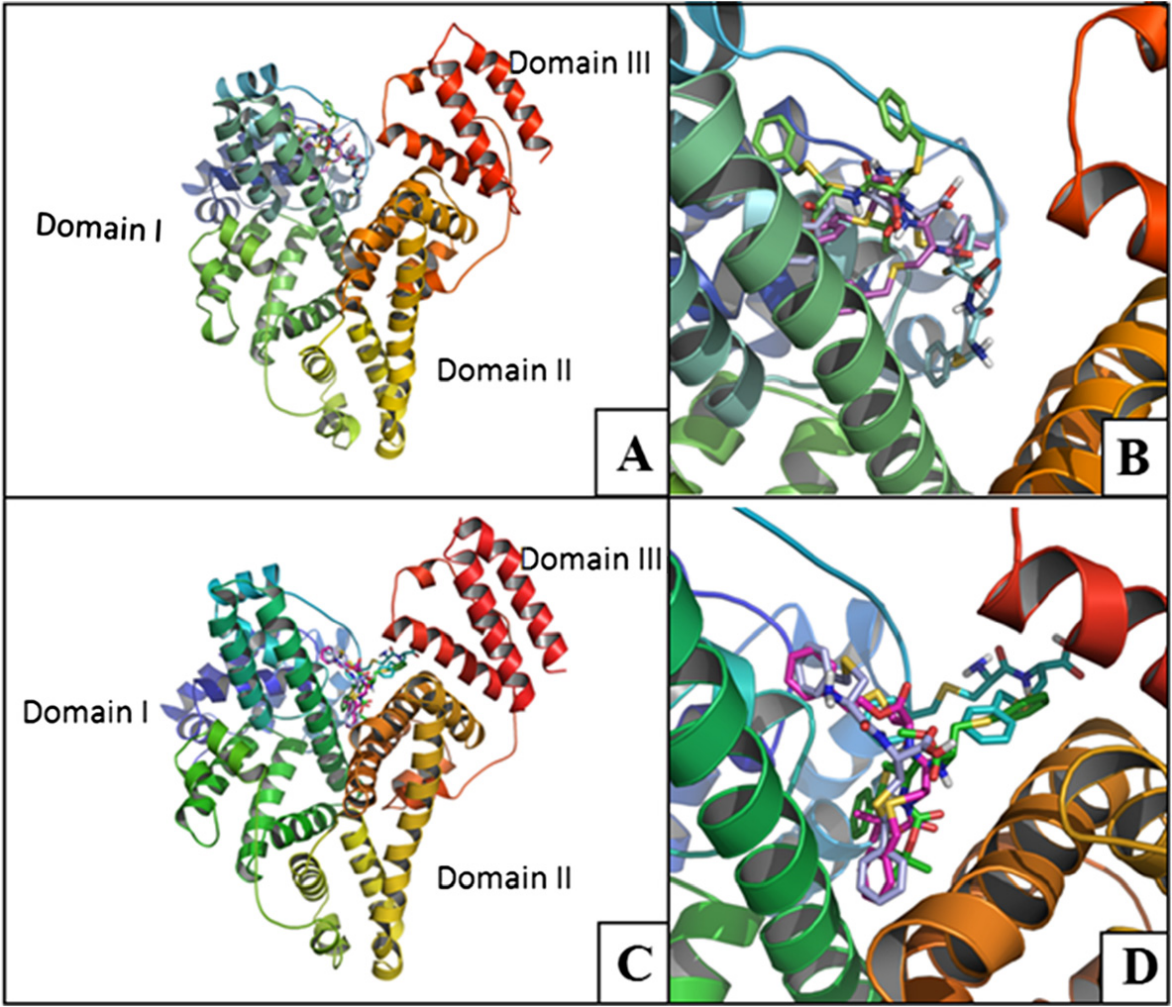
**Ribbon representation of HSA (A) and BSA (C) docked with best binding modes of all ligands along with the close up views, (B) and (D) respectively.** Ribbons are colored in rainbow; blue to red encompassing N-terminal to C-terminal of the proteins. 1**A**, 1**B**, 1**C** and 1**D** are shown in stick models colored in green, magenta, light blue and cyan respectively. In case of HSA best binding modes are found to be in the domain I and for BSA best binding modes clustered in between the domain I and III.

Computational chemistry and molecular modeling studies. BSA and HSA are the major carrier proteins of the serum. These proteins bind to a variety of small molecules, mostly non-specifically, and can strongly affect the way they are delivered through the body. Fluorescence perturbation experiments show that the compounds bind very well with serum albumins, which is also corroborated by docking experiments. Docking of a ligand into a protein binding site and estimating the binding affinity of the resulted complex allow understanding the interaction pattern of a small molecule at the binding site. This information provides vital clues to design structure-based drug molecules. Docking analysis in the current investigation carried out to theoretically evaluate the ability of the compounds to bind serum albumins and the binding site of the receptor. Negative binding energy for the docked conformations (Figure 7A and 7C) indicates that the binding was thermodynamically favourable. Binding free energies for the best docked conformations are listed in Table 3. Here, each docking experiment was a composite of 100 independent iterations producing hundred best docked conformations all of which were arranged according to their binding energy in Figure 7A and 7C. In all the cases, the low energy binding modes indicate thermodynamically favourable interaction. Binding of 1C and 1D with the serum albumins appears to be slightly better than that of 1A and 1B. However, no such narrow discrimination could be established from our experimental results. Specificity of binding can be shown in terms of reproducibility of the docked conformation of the compounds. The docked conformations within 2 Å standard deviation and 0.5 kcal mol^-1^ energy tolerance levels were grouped together into a cluster. Despite of the thermodynamically favorable interaction docking outcomes for each experiment showed no cluster formation (Figure 7B and 7D). Thus, the lack of reproducibility of the docked conformations within 2 Å standard deviation in space, suggests that the binding was nonspecific in nature. The best docked conformations are shown in Figure 8. For HSA the best docked conformation show that these compounds bind in the domain I and in case of BSA they bind between domain I and III. Serum albumins have many fatty acid binding sites. Seven such sites are reported for HSA, of which one site lies in domain I [34].

**Table 3:** Thermodynamic parameter of binding as obtained from molecular docking simulation experiments

**Compounds**	**Binding free energy (kcal mol**^**-1**^**)**	
	**HSA**	**BSA**
**1A**	−4.84	−4.22
**1B**	−4.55	−3.80
**1C**	−5.02	−4.67
**1D**	−4.90	−5.18

Domain I also binds to other drug molecules such as, 2,3,5-triiodobenzoic acid [35]. Small molecules are also reported to bind in to a site in between the domain I and III of HSA [34]. The major contributing forces involved in the binding of these compounds with serum albumins are hydrogen bonding (Additional file 1: Figure S4 and Additional file 1: Figure S5), hydrophobic interaction and van der Waals attraction.

## Conclusion

In conclusion, in this work, we have synthesized four dipeptides made of cystine amino acid (both protected and unprotected form) and studied their interaction with BSA and HSA. Routine solution phase synthesis was employed to prepare these peptides. The cell viability of these compounds was quantified by MTT assay. They show anticancer activity in sub micro molar range. The phase contrast images show massive cell death. The interaction between these compounds with HSA and BSA was investigated by employing different spectroscopic techniques (fluorescence and CD spectroscopy). Fluorescence study indicates strong binding of these compounds with both BSA and HSA. The CD results revel that the secondary structure of BSA and HSA were very slight affected upon interaction with these compounds. The molecular modeling studies show that the binding of these compounds with BSA and HSA are thermodynamically favorable and no cluster formation occurs, which suggest that the bindings are nonspecific in nature. Although detail mechanistic studies of anticancer properties of these molecules are still going on, the initial results indicate DNA intercalation (Additional file 1: Figure S6) may be responsible for the cell death. Further studies in this aspect are going on in our laboratory and the results will be published in due course of time.

## Competing interests

The authors declare that they have no competing interests.

## Authors' contributions

BB, SKP and UP and NCM conceived and designed the experiments. SKP and UP performed the experiments. All the authors analyzed the data. SKP and UP drafted the manuscript. All authors read and approved the final manuscript.

## Authors' information

Biswadip Banerji achieved the following in his academic years: M.Sc. in Chemistry, University of Calcutta, Kolkata, India; Ph.D., Indian Institute of Technology, Kanpur, India; Postdoctoral Research Fellow from Oxford Centre for Molecular Science & Chemistry Research Laboratory, Oxford University, UK; and Postdoctoral Research Fellow from the School of Chemical and Life Sciences, Institute of Chemical & Engineering Sciences-Agency for Science, Technology and Research (ICES-A*STAR), Singapore. He was the Team Leader at Chembiotek, Kolkata, India. He is a Senior Scientist from the Indian Institute of Chemical Biology, Kolkata, India. His research area interests cover smart nanobiomaterials, peptide based drug designing, self assembly of biomaterials and natural product derived hybrid scaffolds and its application in therapeutics.

Sumit Kumar Pramanik obtained his B.Sc. in chemistry from Vidyasagar University, India. He earned his M.Sc. in applied chemistry from Bengal Engineering and Science University, Shibpur, India. He is a Ph.D. Student from the Chemistry Division, Indian Institute of Chemical Biology, Kolkata, India. His research area interests include nanobiomaterials and peptide based drug design and biophysical chemistry.

Uttam Pal earned his B.Sc. in Physiology from the Presidency College, Kolkata, India. He is a M.Sc. degree holder of Biophysics and Molecular Biology from University of Calcutta, Kolkata, India. He is a Ph.D. Student from the Structural Biology and Bioinformatics Division, Indian Institute of Chemical Biology, Kolkata, India. His research area covers structural biology and bioinformatics.

Nakul Chandra Maiti achieved M.Sc. in Chemistry, University of Calcutta, Kolkata, India; Ph.D. From Tata Institute of Fundamental Research, Mumbai, India; Postdoctoral JSPS visiting scientist, Institute for Molecular Science, Japan; Postdoctoral Senior Research Associate, Biochemistry, Case, Cleveland, Ohio, USA; Postdoctoral Research Associate/lecturer, California State University, Los Angeles, USA. He is a Senior Scientist from the Indian Institute of Chemical Biology, Kolkata, India. His research area interests cover structure based amyloid research, structural aspects and in-vitro behavior of natively unfolded proteins and peptides those are linked to human diseases, applications of NMR, fluorescence and Raman spectroscopy to biological systems, computational biochemistry and bioinformatics.

## Supplementary Material

Additional file 1 contains the detailed synthetic procedure and characterization data of these molecules, computational method for partition coefficient (cLogP), detailed mathematical background of fluorescence study, Figures S1-S6, and Table S1. Click here for file
