# Supplementary material for: Potent anticancer activity of cystine-based dipeptides and their interaction with serum albumins
Source: Chem Cent J. 2013 May 24;7:91. doi: 10.1186/1752-153X-7-91 (PMC3680302; doi:10.1186/1752-153X-7-91)
Supplement: Additional file 1 — contains the detailed synthetic procedure and characterization data of these molecules, computational method for partition coefficient (cLogP), detailed mathematical background of fluorescence study, Figures S1-S6, and Table S1. [file 1752-153X-7-91-S1.docx]

Supplementary Information

**Potent anticancer activity of Cystine based dipeptides and Their Interaction with Serum Albumins**

BiswadipBanerji^*^, Sumit Kumar Pramanik, Uttam Pal, Nakul Chandra Maiti.

Methods

Boc-S-benzyl-L-Cysteine from Across Organics, Boc-D-Cys(Bzl)-OH from Sigma Aldrich, S-benzyl-L-Cysteine from Alfa Aesarwere purchased and used without further purification. Solvents were freshly distilled by standard procedures prior to use. Flash chromatography was performed on silica gel (Merk, 100-200 mesh) with the indicated eluant. All ^1^H and ^13^C-NMR spectra were recorded on a Bruker 600 MHz spectrometer. For ^1^H NMR, tetramethylsilane (TMS) served as internal standard (δ = 0) and data are reported as follows: chemical shift, integration, multiplicity (s = singlet, d = doublet, t = triplet, q = quartet, m = multiplet) and coupling constant(s) in Hz. High resolution Mass spectra were obtained on a Jeol MS station 700.

1. **General Procedure to synthesize these dipeptides:**





**Scheme 1** Reagent and conditions: (a) SOCl_2_, MeOH, 0^o^C to rt 4 hrs

To a stirred solution of compound 1 (1.0 equiv.) in methanol was added SOCl_2_ (3.0 equiv.) at 0^o^C. The reaction was then continued at room temperature for 4 hours. The reaction mass was then concentrated to dryness to afford 2, which was carried out to the next step without further purification.





**Scheme 2** Reagent and conditions: (a) EDC.HCl (1.5 equiv.), HOBT (1.2 equiv.), TEA (3 equiv.), 0^o^C to rt, 7.0 hrs.

To a stirred solution of compound 2 (1 equiv.) and compound 3 (1.2 equiv.) in dry THF was added EDC.HCl (1.5 equiv.), HOBT (1.2 equiv.) and triethyl amine (3 equiv.) at 0^o^C. The reaction was then continued at room temperature for 7 hours. The reaction mixture was concentrated to dryness, and the residue was purified by column chromatography (hexane/ ethyl acetate) to afford 4. Two different stereoisomers of 4 were named as 1A and 1B.


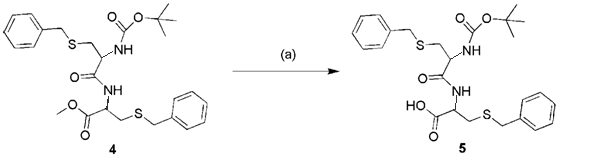


**Scheme 3** Reagent and conditions: (a) Lithium hydroxide (1.5 equiv.), MeOH-water (5:1), rt, 1.5 hrs.

To a stirred solution of compound 4 (1 equiv.) in MeOH-water (5:1) was added lithium hydroxide (1.5 equiv.) at 0^o^C. The reaction was then continued at room temperature for 1.5 hours and monitored by TLC. MeOH was evaporated and the solution was then neutralized by using 1 M HCl solution and the compound was extracted with ethyl acetate. The ethyl acetate layer was then concentrated to dryness to afford 5, which was carried out to the next step without further purification.


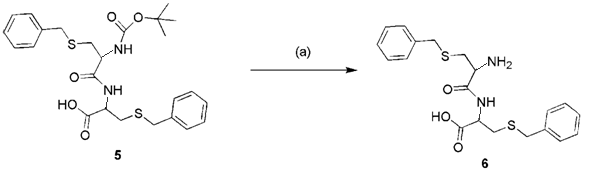


**Scheme 4** Reagent and conditions: (a) 4(M) HCl in 1,4dioxane, 0^o^C to rt, 2.0 hrs.

To a stirred solution of compound 5 (1.0 equiv.) was added 4 (M) HCl in 1,4-dioxane (5.0 equiv.) at 0^o^C. The reaction was then continued at room temperature for 4 hours. The reaction mixture was worked up with ethyl acetate and water. The ethyl acetate was concentrated to dryness, and the residue was purified by column chromatography (chloroform / methanol) to afford 6. Two different stereoisomers of 6 were named as 1C and 1D.

1. **Characterization Data.**

**For compound 1A:**^1^H NMR (600 MHz, CDCl_3_): δ (in ppm) 1.45 (9 H, s), 2.92-2.74 (4 H, m), 3.69 (2 H, s), 3.73 (3 H, s), 3.75 (2 H, s), 4.26 (1 H, b s), 4.77-4.74 (1 H, m) 5.26 (1 H, b s), 7.03 (1 H, b s), 7.25 - 7.22 (2 H, m) 7.27 (1 H, m), 7.29 – 7.28 (3 H, m), 7.31 - 7.30 (3 H, m), 7.34 – 7.33 (2H, m); ^13^C NMR (150 MHz, CDCl_3_): δ (in ppm) 28.30, 33.24, 33.58, 36.46, 36.56, 51.82, 52.65, 80.61, 127.21, 127.24, 128.57, 128.60, 128.91, 129.01, 13.56, 137.84, 170.53, 170.68; HRMS (EI+): m/z Calcd for C_26_H_34_N_2_O_5_S_2_ (M)^+^ 518.1909, Found: m/z 518.1909.

**For compound 1B:**^1^H NMR (600 MHz, CDCl_3_): δ (in ppm) 1.43 (9 H, s), 2.89-2.76 (4 H, m), 3.68 (2 H, s), 3.71 (2 H, s), 3.74 (2 H, s), 4.32 (1 H, b s), 4.74-4.77 (1 H, m) 5.35 (1 H, b s), 7.24- 7.22 (2 H, m), 7.25 - 7.22 (2 H, m) 7.27 (1 H, m), 7.29 – 7.28 (3 H, m), 7.31- 7.30 (3 H, m), 7.34 – 7.33 (2H, m); ^13^C NMR (150 MHz, CDCl_3_): δ (in ppm) 28.30, 33.24, 33.58, 36.46, 36.56, 51.82, 52.65, 80.69, 127.21, 127.24, 128.57, 128.60, 128.91, 129.01, 13.56, 137.84, 170.53, 170.68;HRMS (EI+): m/z Calcd for C_26_H_34_N_2_O_5_S_2_ (M)^+^ 518.1909, Found: m/z 518.1904.

**For compound 1C:**^1^H NMR (600 MHz, D_2_O): δ (in ppm) 2.69-2.81 (4H, m), 3.76-3.74 (4H, m), 4.25-4.26 (1H, m), 4.49-4.52 (1H, m), 7.21-7.25 (2H, m), 7.28-7.32 (8H, m); ^13^C NMR (150 MHz, D_2_O): δ (in ppm) 32.33, 33.68, 35.17, 35.37, 52.14, 53.57, 126.77, 126.92, 128.31, 128.37, 128.91, 138.01, 138.47, 170.87, 170.93; HRMS (EI+): m/z Calcd for C_20_H_24_N_2_O_3_S_2_ (M)^+^404.1228, Found: m/z 404.1220.

**For compound 1D:**^1^H NMR (600 MHz, D_2_O): δ (in ppm) 2.67-2.82 (4H, m), 3.73-3.76 (4H, m), 4.28-4.29 (1H, m), 4.51-4.54 (1H, m), 7.20-7.24 (4H, m), 7.25-7.31 (6H, m); ^13^C NMR (150 MHz, D_2_O): δ (in ppm) 33.16, 33.50, 35.20, 35.41, 52.14, 53.69, 126.82, 126.92, 128.35, 128.38, 128.96, 138.08, 138.47, 170.87, 170.94; HRMS (EI+): m/z Calcd for C_20_H_24_N_2_O_3_S_2_ (M)^+^ 404.1228, Found: m/z 404.1223.

**Computation of partition coefficient (cLogP).**Theoretical partition coefficient in toluene/water for the compounds were calculated by Molecular Property Explorer at Organic Chemistry Portal (http://www.organic-chemistry.org/).

**Fluorescence study.**Considering a protein P has a single binding site for a quencher Q, at saturation, one mole of P binds with one mole of Q to form a complex PQ.

$P+Q\Leftrightarrow PQ$ (1)

The dissociation constant K_d_ is the reciprocal of equilibrium constant (K) in terms of general chemistry or the affinity constant (K_a_) in biology. For the association reaction as stated above $K_{d}$is defined as

$K_{d}=\frac{\left[ P \right]\left[ Q \right]}{\left[ PQ \right]}$ (2)

Stern-Volmer (S-V) quenching constant, which is the equilibrium constant of a quenching reaction, gives the value of dissociation constant when inversed. Equation (2) shows that the fraction of quencher bound is affected by the [P] and [Q]. In a fluorescence quenching experiment, the number of moles of ligand bound to one mole of protein can be measured as follows:

$\frac{\left[ Q \right]_{bound}}{\left[ P \right]_{total}}=\frac{\Delta F}{\Delta F_{max}}=\frac{\left[ PQ \right]}{\left[ P \right]+\left[ PQ \right]}$ (3)

Combining the equations (2) and (3) we get the renowned *Langmuir isotherm*:

$\frac{\Delta F}{\Delta F_{max}}=\frac{\left[ Q \right]}{K_{d}+\left[ Q \right]}$ (4)

Plotting ΔF against the quencher concentration and fitting a non-linear regression the value of K_d_ and the ΔF_max_ can be obtained (Fig. S1 left panels). Taking reciprocal on both sides allow the data to be plotted on a straight-line graph (which is called a *double-reciprocal* plot):

$\frac{1}{\Delta F}=\frac{K_{d}}{\Delta F_{max}}\cdot\frac{1}{\left[ Q \right]}+\frac{1}{\Delta F_{max}}$ (5)

K_d_ and the ΔF_max_ can be obtained from this double reciprocal plot by simple regression analysis (Fig. S1 left panels). If we further modify the equation (5) we get

$\frac{F_{0}}{\Delta F}=\frac{F_{0}K_{d}}{\Delta F_{max}}\cdot\frac{1}{\left[ Q \right]}+\frac{F_{0}}{\Delta F_{max}}$ (6)

The ratio ΔF_max_ to F_0_ is nothing but the fraction of accessible fluorescence to the quencher and can be denoted as *f*. If we further replace the K_d_ in this equation with S-V quenching constant or the affinity constant, K_a_, we arrive at the modified S-V equation.^24^

$\frac{F_{0}}{\Delta F}=\frac{1}{fK_{a}\left[ Q \right]}+\frac{1}{f}$ (7)

The quenching data was analysed using equations (4), (5) and (7) to obtain different parameters. The data nicely fitted with the mathematical models of the hypothetical system that assumes the mode of binding.


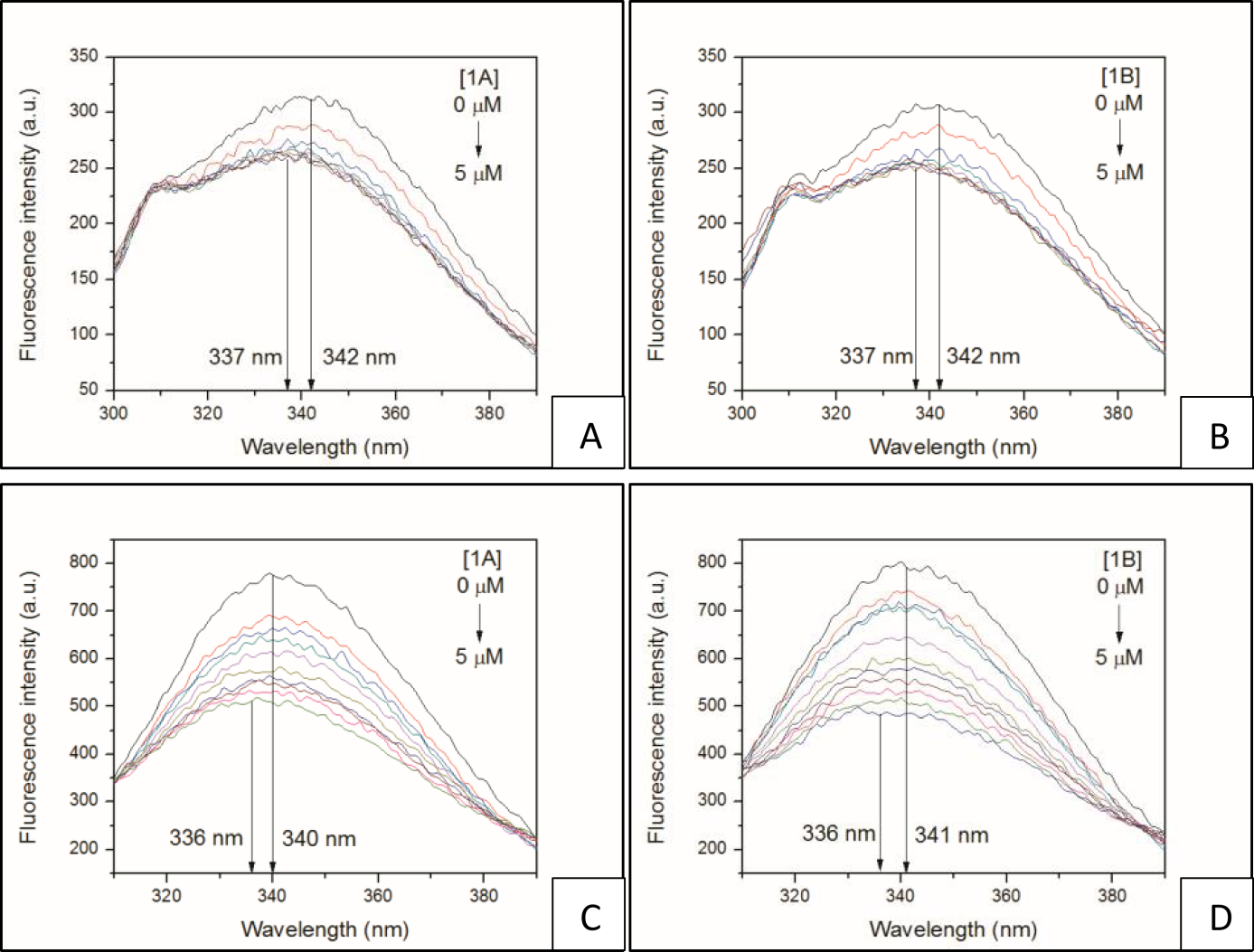


**Fig. S1** Effect of the compounds on the intrinsic fluorescence of serum albumins. Here A and B are Emission spectra of HSA as a function of compound 1A and 1B respectively with concentration varying from 0 µM to 5 µM (10 steps). Figure C and D are Emission spectra of BSA as a function of compound 1A and 1B respectively with concentration varying from 0 µM to 5 µM (10 steps). About 4 to 6 nm blue shift in emission maximum was observed. Excitation maximum, 280 nm; excitation and emission slit 5 nm each.


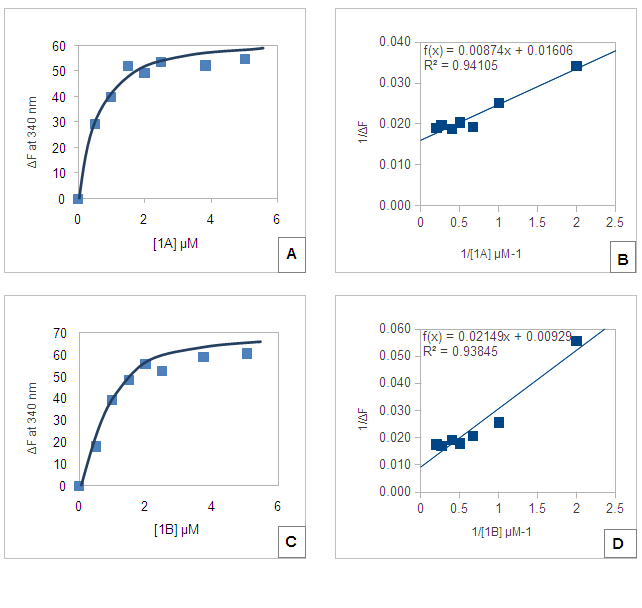


**Fig. S2a** Calculation of binding constants with HSA forms fluorescence perturbation experiments. (**A**) and (**B**) for compound 1A. (**C**) and (**D**) compound for 1B.


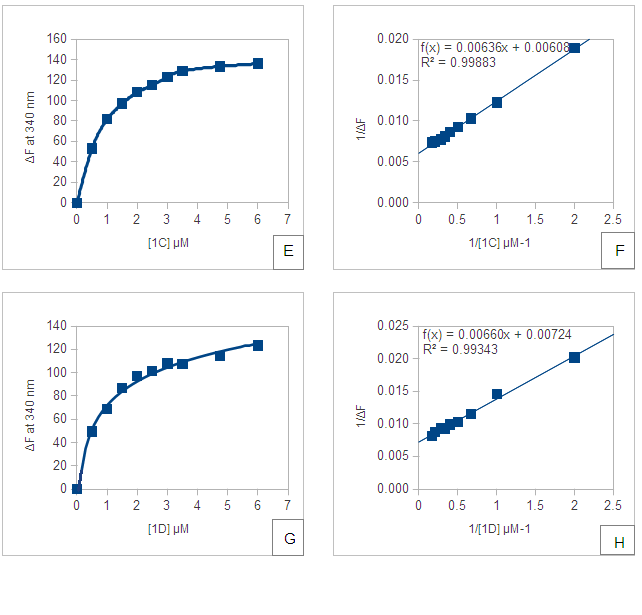


**Fig.S2b** Calculation of binding constants with HSA from fluorescence perturbation experiments. (**E**) and (**F**) for compound 1C. (**G**) and (**H**) for compound 1D.


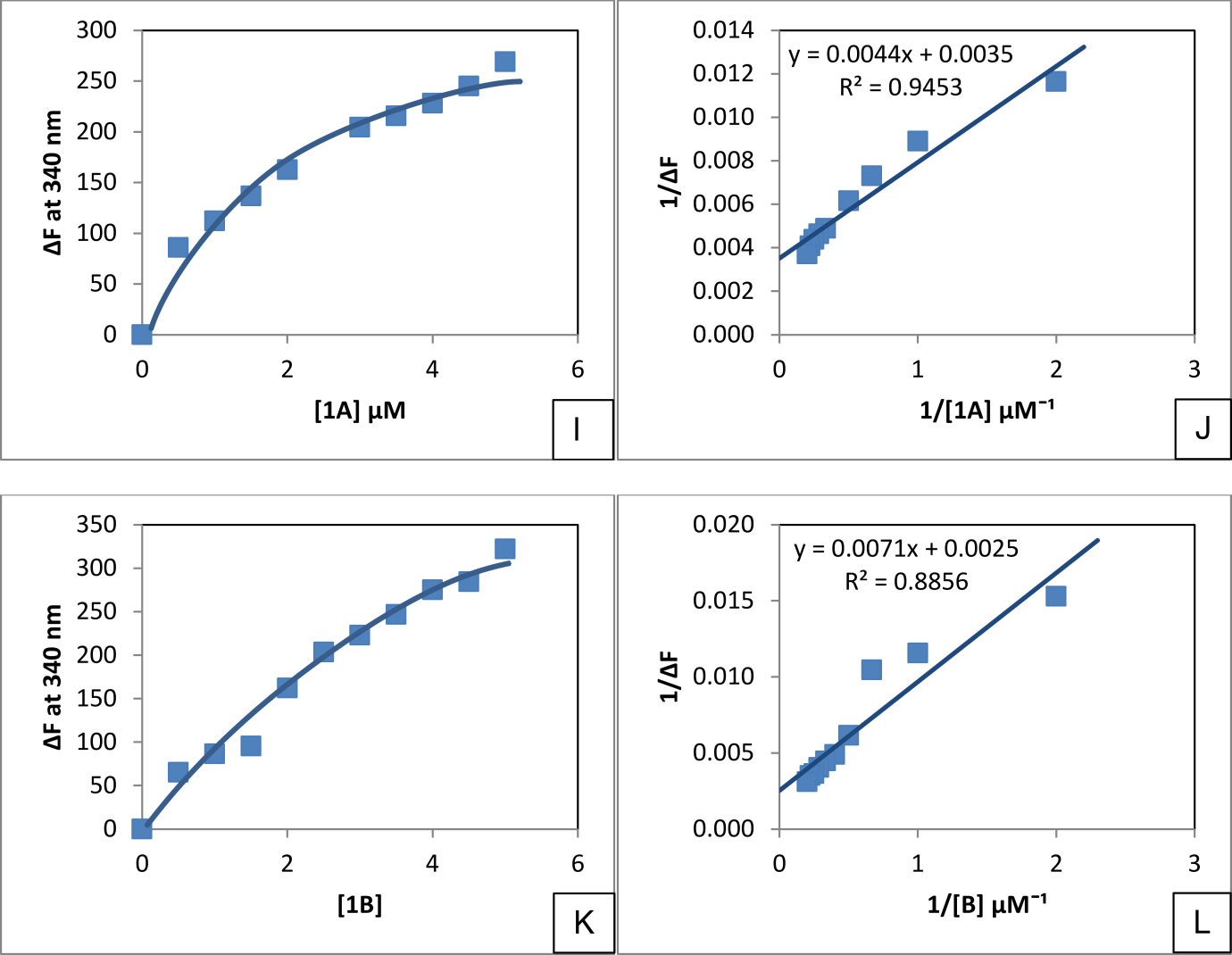


**Fig. S2c** Calculation of binding constants with BSA from fluorescence perturbation experiments. (**I**) and (**J**) for compound 1A. (**K**) and (**L**) for compound 1B.


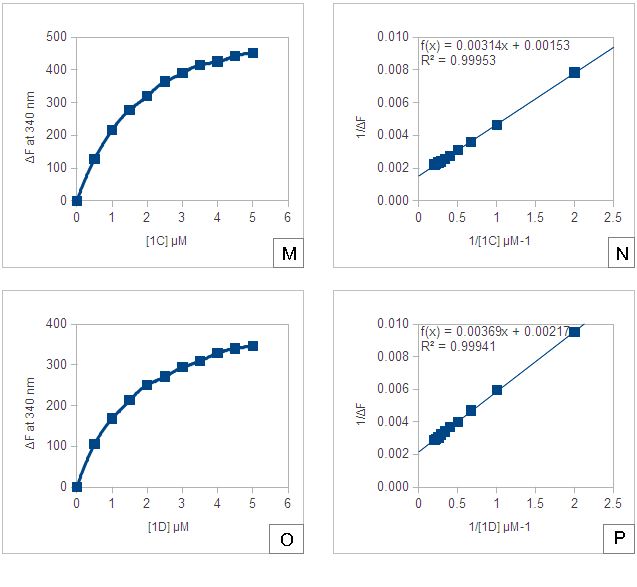


**Fig. S2d** Calculation of binding constants with BSA from fluorescence perturbation experiments. (**M**) and (**N**) for compound 1C. (**O**) and (**P**) for compound 1D.

**Table S1: Temperature dependence of Stern-Volmer quenching constant for BSA fluorescence**

| **Temperature (K)** | Stern-Volmer quenching constant for compound 1D |
| --- | --- |
| **293** | 6.65×10^5^ |
| **298** | 5.90×10^5^ |
| **303** | 5.12×10^5^ |
| **308** | 4.33×10^5^ |


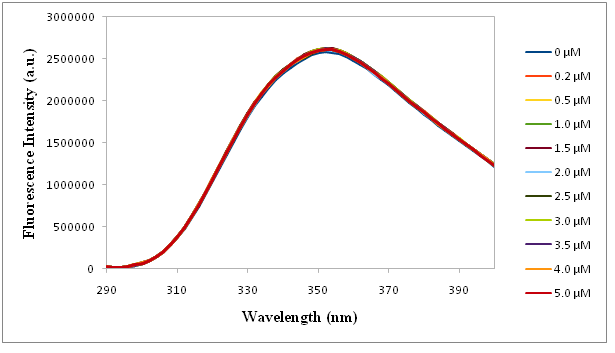


**Fig.S3** Quenching of free tryptophan fluorescence with compound 1D. Tryptophan concentration was 0.5 μM (in Tris-HCl buffer, pH 7.5) and the quencher (dipeptides) concentration was varied from 0.2—5.0 μM. The spectra was collected at 25 ºC in a PTI QuantaMaster™ 40 spectrofluorimeter. Both the emission and excitation slits were 5 nm. Excitation wavelength was 280 nm. No change in the fluorescence intensity due to the addition of the quencher was observed.

**Closeup view of the docked conformations.**


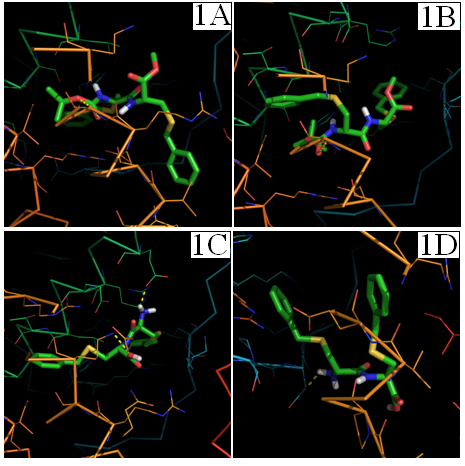


**Fig. S4** Close up view of the docked conformations of 1A to 1D with BSA (Fig. 1A, 1B, 1C, 1D are for compounds 1A, 1B, 1C, 1D respectively) to show the involvement of hydrogen bonding in the interactions. Hydrogen bonds are shown by yellow dotted lines. Compounds are shown in stick model and BSA as ribbon diagram. Interacting side chains of BSA are also shown in line representation.


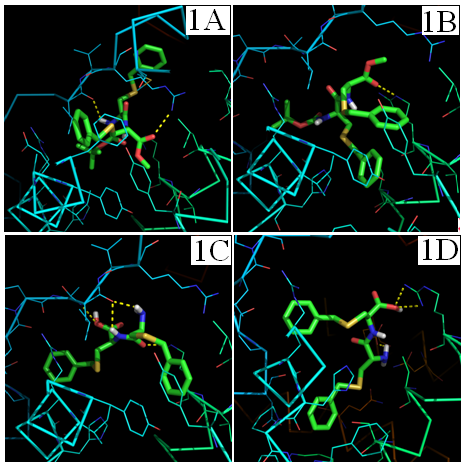


**Fig. S5**Close up view of the docked conformations of 1A to 1D with HSA (Fig. 1A, 1B, 1C, 1D are for compounds 1A, 1B, 1C, 1D respectively) to show the involvement of hydrogen bonding in the interactions. Hydrogen bonds are shown by yellow dotted lines. Compounds are shown in stick model and HSA as ribbon diagram. Interacting side chains of HSA are also shown in line representation.

***In silico* DNA binding study.**

**
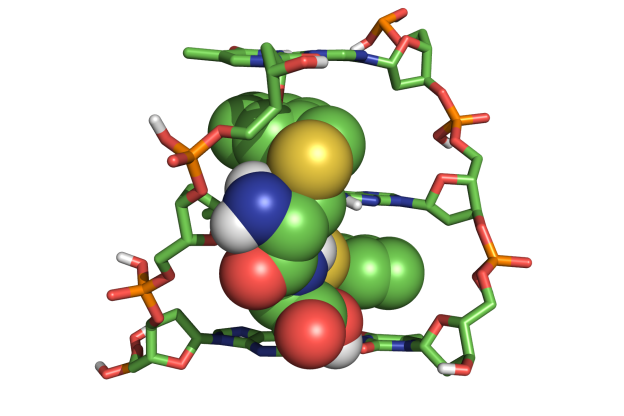

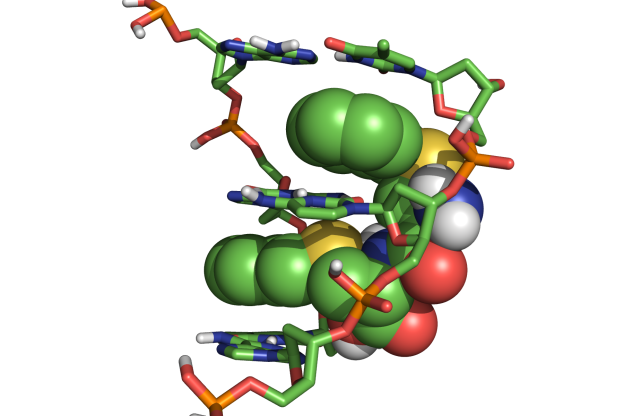

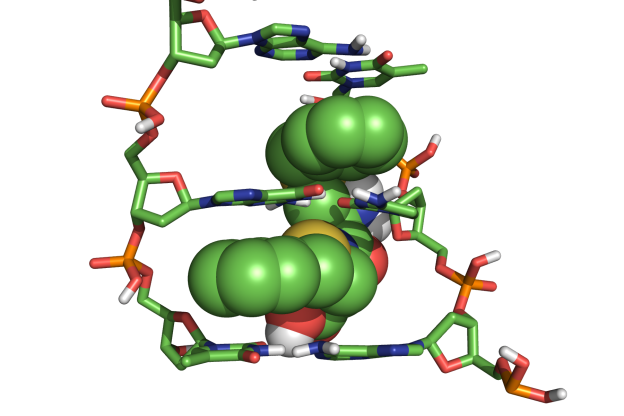
**

90º

90º

**Fig. S6**Molecular docking simulation of 1Dwith B-DNA showedthat the aromatic rings in 1D intercalated into the base-pairs of B-DNA. The binding free energy was found to be -7.35 kcal mol^-1^ indicating a thermodynamically favorable interaction. DNA is showm in stick model and 1D is shown in space filling model. Standard color representation for the elements was followed.
